# Neuronal intranuclear inclusion disease with initial manifestation of intractable nausea and vomiting responsive to corticosteroids: a case report

**DOI:** 10.3389/fimmu.2026.1782547

**Published:** 2026-03-18

**Authors:** Long Luo, Ling Zhu, Yong Liang, Ying Yuan, Lei Chen, Weiwen Peng, Gao Yang, Ronghe Yang

**Affiliations:** 1 Department of Neurology, The Central Hospital of Xiangtan (The Affiliated Hospital of Hunan University), Xiangtan, Hunan, China; 2 Department of Neurology, The Central Hospital of Shaoyang, Shaoyang, Hunan, China

**Keywords:** corticosteroid, immune, inflammatory, neuronal intranuclear inclusion disease, NIID, vomit

## Abstract

Neuronal intranuclear inclusion disease (NIID) can initially present with gastrointestinal symptoms as the sole or primary manifestation for decades before neurological signs emerge. We report the case of a 61-year-old woman with a 22-year history of drug-refractory, recurrent nausea and vomiting. Previous extensive gastrointestinal evaluations were unremarkable, and conventional therapies proved ineffective. The diagnosis of NIID was established on the basis of the following key findings: diffusion-weighted imaging revealed a characteristic “crown-like” hyperintensity at the corticomedullary junction; genetic analysis revealed a GGC repeat expansion in the *NOTCH2NLC* gene, with no abnormalities detected in *FMR1*; and skin biopsy demonstrated p62-positive intranuclear inclusions within sweat gland cells. The patient’s symptoms improved markedly with corticosteroid therapy but recurred upon repeated withdrawal. Subsequent initiation of a maintenance corticosteroid regimen resulted in sustained clinical stability, with only occasional mild symptom fluctuations over a six-month follow-up period. This case highlights that vomiting can be a prominent and early clinical feature of NIID. The condition should be considered in the differential diagnosis of patients with unexplained, refractory nausea and vomiting, for whom long-term corticosteroid therapy may represent a viable treatment strategy.

## Introduction

Neuronal intranuclear inclusion disease (NIID) is named for its pathological hallmark: eosinophilic intranuclear inclusions within neurons. Clinically, the disease presents with remarkable heterogeneity, including cognitive impairment, movement disorders, paroxysmal symptoms, muscle weakness, and autonomic dysfunction (1, 2). These features frequently overlap, resulting in mixed phenotypic presentations. Although the overall disease course is slowly progressive, patients may experience acute, subacute, or episodic symptomatic exacerbations (3). Epidemiologically, early European cases often reported childhood or adolescent onset, whereas more recent large cohort studies from East Asia indicate a predominance of middle-aged and elderly patients; familial cases generally present earlier than sporadic cases do, with a reported male-to-female ratio of approximately 1:2 (2, 4, 5).

Gastrointestinal symptoms represent a common systemic manifestation of NIID, affecting approximately 64.7% of patients with varying severity (6). The clinical spectrum is broad and may include nausea, vomiting, constipation, gastroenteritis, pseudo-obstruction, achalasia, and abnormal liver function (6, 7). Among these, nausea and vomiting are common, with prevalence rates ranging from 14.6% to 43.1% (1, 4, 6). In rare cases, nausea and vomiting may serve as the primary or even sole clinical manifestations during the early stages of NIID (7–10). This atypical presentation pattern often leads to misdiagnosis or delayed diagnosis. Consequently, NIID should be considered in the differential diagnosis of unexplained, treatment-refractory nausea and vomiting.

NIID is a refractory and progressive neurological disorder characterized by persistent functional decline throughout its clinical course (11). Current treatment options remain limited, posing significant challenges for long-term disease control. Published observations suggest that corticosteroids may alleviate certain symptoms, such as encephalitis-like episodes (4, 12–14) and some gastrointestinal manifestations (7, 14–16). However, its efficacy against other core disease features, such as muscle weakness and peripheral neuropathy, is often unsatisfactory (15). These heterogeneous treatment responses underscore the necessity for further research to better define the precise indications and optimize therapeutic protocols for corticosteroid administration in NIID patients.

## Case presentation

**Figure 1:**
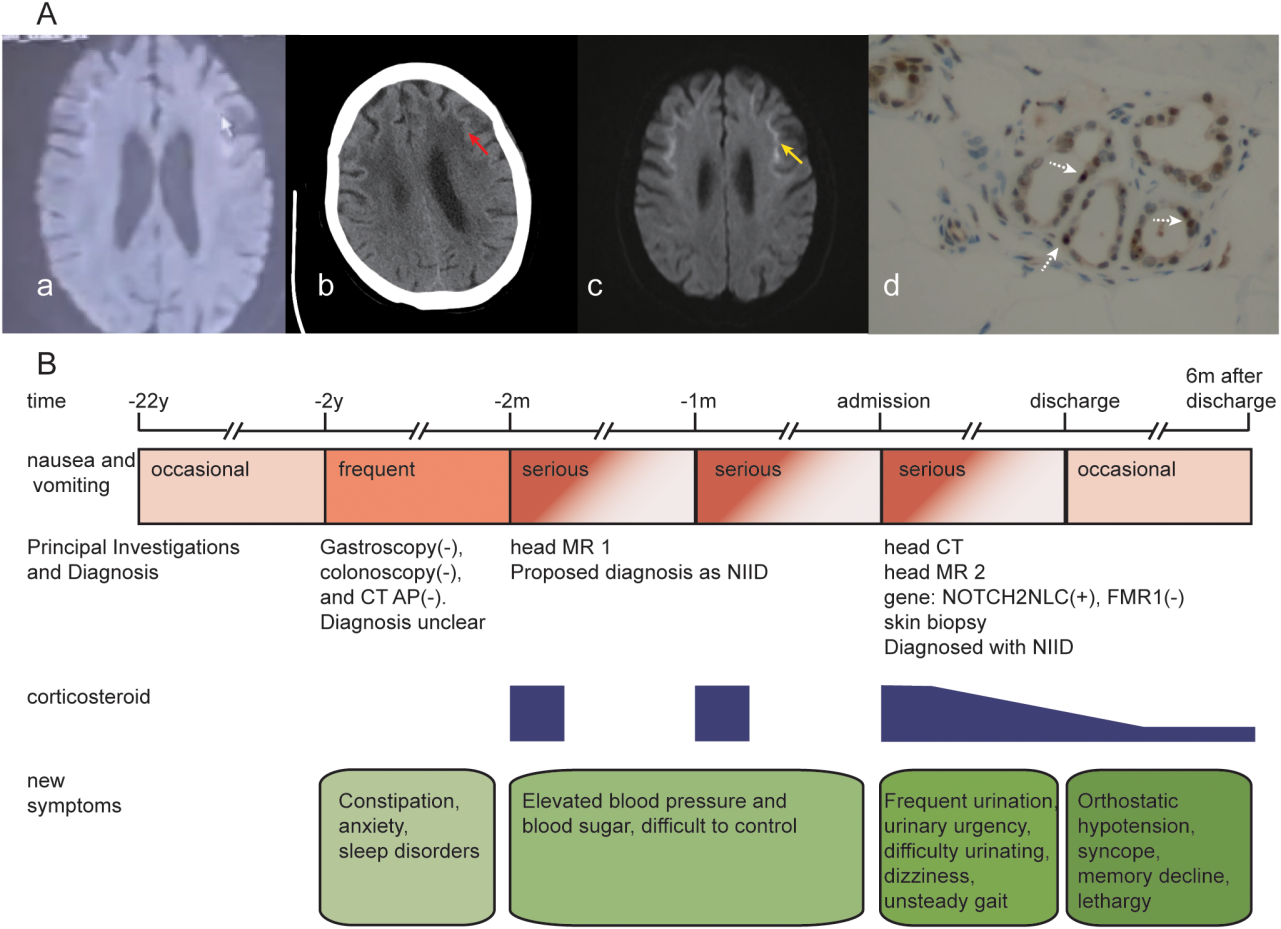
**(A)** summarizes the neuroimaging and histopathological features of the patient: (a) axial DWI from a local hospital two months before admission shows hyperintense white matter lesions at the corticomedullary junction; (b) non-contrast head CT performed after admission to our hospital shows abnormal white matter signals in the same region; (c) follow-up axial DWI after admission to our hospital demonstrates persistent corticomedullary junction hyperintensity; (d) skin biopsy of the right leg shows p62-positive intranuclear inclusions within sweat gland cells. **(B)** illustrates the timeline of clinical events, with abbreviations: CT AP = abdominal-pelvic computed tomography; NIID = neuronal intranuclear inclusion disease.

A 61-year-old woman with a 22-year history of recurrent nausea and vomiting that had progressively worsened over the past two years was admitted to our hospital. Initially, she experienced episodes of dry retching every 3–4 months, each lasting 1–4 days, often triggered by emotional stress. Over the past two years, the frequency has increased every 2–3 months, accompanied by vomiting of gastric contents, constipation, anxiety, psychological distress, and sleep disturbances. Previous gastroenterological evaluations, including abdominopelvic CT and gastrointestinal endoscopy, revealed no significant abnormalities. Symptoms persisted despite treatment with prokinetic and laxative agents. Two months prior to admission, she presented to a local hospital with acute severe nausea and vomiting that persisted for one week without improvement. During this episode, her blood pressure increased sharply from a baseline of 130/80 mmHg to 170–220/90–120 mmHg, accompanied by elevated blood glucose levels (increasing from the upper-normal range to 15–26 mmol/L). Diffusion-weighted imaging (DWI) revealed abnormal white matter signals at the corticomedullary junction (**Figures 1A, a**), leading to a provisional diagnosis of NIID. Symptoms resolved completely within two days after initiating dexamethasone (10 mg once daily) but recurred upon discontinuation after discharge. One month before admission, she received the same dexamethasone regimen at another hospital, which again resulted in rapid symptom resolution followed by recurrence after cessation. She was subsequently referred to our hospital for further evaluation and management.

At the current presentation, the patient reported newly emerged symptoms, including urinary urgency, frequency, dysuria, dizziness, and unsteady gait without directional predominance. She had a 5-year history of hypertension, which was well controlled with irbesartan-hydrochlorothiazide (one tablet daily). There was no reported family history of inherited neurological disorders. Neurological examination revealed normal muscle strength (MRC grade 5), tone, and deep tendon reflexes. Finger-to-nose and heel-to-shin tests were normal, and the Romberg test was negative. Gait was wide-based, with the inability to perform tandem walking and marked difficulty in turning. No miosis, significant vision loss, visual field defects, or dysarthria was observed. Neuropsychological assessment revealed the following: MoCA score 28/30 (normal ≥26), MMSE 27/30, FAB 18/18 (adult mean approximately 16), HAMA 20 (cutoff 14), HAMD 12 (suggesting a possible depressive state), and Barthel Index 80 (indicating mild functional dependency). Laboratory investigations—including urinalysis, stool studies, coagulation profile, D-dimer, homocysteine, renal and hepatic function, glucose, lipid panel, cardiac enzymes, electrolytes, C-reactive protein, HbA1c, catecholamines, infectious disease screening, thyroid function and antibodies, vitamin B12, folate, and antinuclear antibodies—were all within normal limits. The plasma lactate concentration was 2.45 mmol/L, and the white blood cell count was 9.37 × 10^9^/L (**Supplementary Table 1**). The electrocardiogram was unremarkable. Lumbar puncture was declined by the patient. Head CT revealed subcortical white matter lesions (**Figures 1A, b**). DWI revealed characteristic “crown-like” hyperintensity in subcortical regions (**Figures 1A, c**). Video-electroencephalography revealed mild delta activity over the right frontotemporal and midline frontal regions. Genetic testing confirmed a GGC repeat expansion in the *NOTCH2NLC* gene, whereas CGG repeats in *FMR1* were normal. Skin biopsy from the right leg revealed multiple p62-positive intranuclear inclusions within sweat gland cells (**Figures 1A, d**). A definitive diagnosis of NIID was established.

The patient was initially treated with intravenous methylprednisolone (80 mg daily), concomitant with management for blood pressure and blood glucose, maintenance of fluid and electrolyte balance, and supplementation with coenzyme Q10. Given her concurrent psychological distress, a psychiatry consultation was obtained. The consulting team advised that pharmacotherapy was an option if clinically indicated but not compulsory. After shared decision-making with the patient and her family, we elected not to initiate anxiolytic or antidepressant medications at that time. This regimen resulted in marked symptomatic improvement within 24 hours and near-complete resolution within three days. Upon discharge, the medication was switched to oral prednisone (60 mg daily), which was tapered by one tablet every 5 to 7 days, eventually maintaining a daily dose of 10 mg. During the six-month follow-up period, episodes of vomiting substantially improved, with only occasional dry retching reported. However, unsteady gait, urinary symptoms, and constipation persisted without significant improvement. New-onset orthostatic hypotension (standing blood pressure <90/60 mmHg) developed, which was associated with one episode of syncope. Progressive memory decline and reduced mental acuity were also noted during follow-up (**Figure 1B**).

## Discussion

Currently, there is no unified diagnostic standard for NIID, and its diagnosis relies on a comprehensive analysis of imaging, genetic, and pathological evidence. The characteristic “crown-like” sign of high signal intensity along the corticomedullary junction on DWI is considered a relatively specific neuroimaging marker (4). Importantly, however, this sign is absent in 3.4–18.2% of NIID patients (1, 2, 4, 17, 18). Furthermore, similar imaging findings can be observed in other neurological disorders, such as Creutzfeldt–Jakob disease, fragile X-associated tremor/ataxia syndrome (FXTAS), and cerebral autosomal dominant arteriopathy with subcortical infarcts and leukoencephalopathy. At the genetic and pathological levels, inconsistencies may arise. The detection of a GGC repeat expansion in the *NOTCH2NLC* gene and the identification of eosinophilic intranuclear inclusions do not always coincide. Some cases present with pathological confirmation but negative genetic testing (19–21), whereas others are genetically confirmed but exhibit atypical pathological features (5, 22). Given the significant clinical and pathological overlap between NIID and FXTAS, genetic testing for the *FMR1* gene is essential for differential diagnosis (23). In the present case, the convergence of the characteristic neuroimaging findings, a confirmed *NOTCH2NLC* GGC repeat expansion with normal *FMR1* gene repeats, and definitive skin biopsy results provided comprehensive diagnostic confirmation of NIID.

In rare instances, gastrointestinal symptoms can serve as the initial or predominant manifestation of NIID (8, 9). This clinical presentation may be attributed to the involvement of peripheral organs, such as the digestive system, preceding central nervous system pathology (2, 6). Histopathological studies have revealed that eosinophilic intranuclear inclusions are widely distributed in both central and peripheral structures governing gastrointestinal function. These include the hypothalamus, autonomic centers, the intermediolateral nucleus, and the gastrointestinal tract itself—such as the esophagus, stomach, gallbladder, and rectum (6–8, 24–26). Concurrently, a loss of neurons in the myenteric and submucosal plexuses has been observed (7, 27). These pathological changes are thought to disrupt immune regulation and secretory functions within the gut, potentially resulting in gastrointestinal symptoms (28). Consequently, NIID should be included in the differential diagnosis when evaluating patients with unexplained or treatment-refractory gastrointestinal symptoms.

Clinical, laboratory, and pathological observations provide multifaceted evidence supporting immune-mediated mechanisms in NIID: First, from the perspective of neuroimaging and therapeutic response, patients presenting with encephalitis-like episodes may exhibit focal cortical edema, diffusion restriction, and gadolinium enhancement (4, 12, 17, 18, 29)—abnormalities typically reversible following corticosteroid therapy (14, 30)—while gastrointestinal symptoms have also demonstrated favorable and sustained responses to corticosteroids across pediatric, adolescent, and adult populations including both familial and sporadic cases (7, 14–16), with additional reports documenting improvements in psychiatric disturbances (14), renal impairment (15), tremor, and memory function (31); second, plasma levels of proinflammatory cytokines (e.g., IL-6 and TNF-α) are significantly elevated in NIID patients compared with healthy controls (32); third, inflammatory cell infiltration has been confirmed in regions adjacent to intranuclear inclusions (6, 14). In the present case, the patient’s gastrointestinal symptoms followed a relapsing-progressive rather than self-limiting course, and we observed a clear withdrawal response (symptom recurrence following two separate corticosteroid tapering attempts) and a positive rechallenge phenomenon (near-complete or complete symptom resolution shortly after re-initiation on two occasions), with sustained symptomatic stability during subsequent maintenance therapy further supporting corticosteroid-responsiveness. A similar dependency has been documented in other clinical contexts. For example, Yadav et al. reported a 50-year-old male NIID patient whose tremor and memory significantly improved after pulse corticosteroid therapy, worsened during dose reduction, and improved again upon retreatment (31). Collectively, these observations suggest that inflammatory processes likely contribute to the pathogenesis of certain clinical manifestations in NIID, and that corticosteroids may exert symptomatic benefits by suppressing this inflammatory component (33). Nevertheless, given that most of the available data are uncontrolled, these findings should be interpreted with caution; furthermore, whether corticosteroids exert disease-modifying effects or merely provide symptomatic relief warrants further investigation.

## Limitations

This study has several limitations. First, gastrointestinal biopsy and cerebrospinal fluid analysis were not performed. Second, inflammatory markers such as IL-6 and TNF-α were not measured. Third, although the patient’s gastrointestinal symptoms showed a favorable response to corticosteroids during the observation period, this therapeutic effect requires further confirmation through long-term follow-up.

## Conclusion

This case highlights that gastrointestinal symptoms may as an early, predominant, or even sole manifestation of NIID. Furthermore, a key therapeutic insight emerged from the observed reproducible pattern of gastrointestinal symptom recurrence following corticosteroid withdrawal and re-improvement upon rechallenge. This pattern, along with the sustained stability achieved with maintenance therapy, suggests a state of corticosteroid responsiveness. The generalizability of this phenomenon, however, requires further validation in larger patient cohorts.

## Patient’s perspective

For over two decades, I mistakenly attributed my recurrent nausea and vomiting to gastrointestinal disorders. Despite numerous tests and medications, no relief was achieved. The turning point came when a brain MRI, a test I had never undergone before, revealed abnormal signals—finally pointing toward a diagnosis: NIID.

The therapeutic effect of corticosteroids was remarkable. During the first severe episode, intractable nausea and vomiting persisted for an entire week and could not be relieved even with complete fasting. However, within just one to two days of corticosteroid administration, my symptoms completely and unexpectedly resolved. During subsequent recurrences, I proactively sought corticosteroid treatment, which again proved effective. I am currently on low-dose maintenance therapy and have not been hospitalized for nausea and vomiting since. Although I have experienced weight gain, the control of my most distressing symptoms has given me great confidence in the treatment. I share this story in the hope of helping other patients who may have similar experiences.

## Data Availability

The datasets presented in this study can be found in online repositories. The names of the repository/repositories and accession number(s) can be found in the article/**Supplementary Material**.

## References

[1] Tian Y Zhou L Gao J Jiao B Zhang S Xiao Q . Clinical features of NOTCH2NLC-related neuronal intranuclear inclusion disease. J Neurol Neurosurg Psychiatry. (2022) 93:1289–98. doi: 10.1136/jnnp-2022-329772, PMID: 36150844 PMC9685690

[2] Tai H Wang A Zhang Y Liu S Pan Y Li K . Clinical features and classification of neuronal intranuclear inclusion disease. Neurol Genet. (2023) 9:e200057. doi: 10.1212/NXG.0000000000200057, PMID: 37090934 PMC10117695

[3] Sone J Mori K Inagaki T Katsumata R Takagi S Yokoi S . Neuronal intranuclear inclusion disease: recognition and update. J Neural Transm (Vienna Austria: 1996). (2021) 128:295–303. doi: 10.1007/s00702-021-02313-3, PMID: 33599827

[4] Sone J Mori K Inagaki T Katsumata R Takagi S Yokoi S . Clinicopathological features of adult-onset neuronal intranuclear inclusion disease. Brain: J Neurol. (2016) 139:3170–86. doi: 10.1093/brain/aww249, PMID: 27797808 PMC5382941

[5] Tian Y Wang JL Huang W Zeng S Jiao B Liu Z . Expansion of human-specific GGC repeat in neuronal intranuclear inclusion disease-related disorders. Am J Hum Genet. (2019) 105:166–76. doi: 10.1016/j.ajhg.2019.05.013, PMID: 31178126 PMC6612530

[6] Chen H Lu L Wang B Cui G Wang X Wang Y . Re-defining the clinicopathological spectrum of neuronal intranuclear inclusion disease. Ann Clin Trans Neurol. (2020) 7:1930–41. doi: 10.1002/acn3.51189, PMID: 32931652 PMC7545592

[7] Barnett JL McDonnell WM Appelman HD Dobbins WO . Familial visceral neuropathy with neuronal intranuclear inclusions: diagnosis by rectal biopsy. Gastroenterology. (1992) 102:684–91. doi: 10.1016/0016-5085(92)90121-E, PMID: 1310083

[8] Schuffler MD Bird TD Sumi SM Cook A . A familial neuronal disease presenting as intestinal pseudoobstruction. Gastroenterology. (1978) 75:889–98. doi: 10.1016/0016-5085(78)90476-6 212342

[9] Okamura S Takahashi M Abe K Inaba A Sone J Orimo S . A case of neuronal intranuclear inclusion disease with recurrent vomiting and without apparent DWI abnormality for the first seven years. Heliyon. (2020) 6:e04675. doi: 10.1016/j.heliyon.2020.e04675, PMID: 32817896 PMC7424193

[10] Liu X Liu X Du Y Lin Y Li C Liu C . A case of recurrent vomiting: extending the spectrum of neuronal intranuclear inclusion disease. Neurol Sci: Off J Ital Neurol Soc Ital Soc Clin Neurophysiol. (2019) 40:2661–4. doi: 10.1007/s10072-019-03986-1, PMID: 31267307

[11] Zhou L Tian Y Zhang S Jiao B Liao X Zhou Y . Characteristics of autonomic dysfunction in neuronal intranuclear inclusion disease. Front Neurol. (2023) 14:1168904. doi: 10.3389/fneur.2023.1168904, PMID: 37388545 PMC10300412

[12] Liang H Wang B Li Q Deng J Wang L Wang H . Clinical and pathological features in adult-onset NIID patients with cortical enhancement. J Neurol. (2020) 267:3187–98. doi: 10.1007/s00415-020-09945-7, PMID: 32535679

[13] Li J Zhang G Zheng J Hu J Li Y . A case report of neuronal intranuclear inclusion disease and literature review. BMC Neurol. (2024) 24:488. doi: 10.1186/s12883-024-03997-2, PMID: 39707256 PMC11660584

[14] Mori K Yagishita A Funata N Yamada R Takaki Y Miura Y . Imaging findings and pathological correlations of subacute encephalopathy with neuronal intranuclear inclusion disease-Case report. Radiol Case Rep. (2022) 17:4481–6. doi: 10.1016/j.radcr.2022.08.084, PMID: 36189161 PMC9519487

[15] Morita K Shinzato T Endo Y Suzuki M Yoshida H Sone J . A case of unusual renal manifestation in a patient with neuronal intranuclear inclusion disease treated with steroids. Clin Case Rep. (2023) 11:e7730. doi: 10.1002/ccr3.7730, PMID: 37564608 PMC10410123

[16] Miyamoto Y Okazaki T Watanabe K Togawa M Adachi T Kato A . First detailed case report of a pediatric patient with neuronal intranuclear inclusion disease diagnosed by NOTCH2NLC genetic testing. Brain Dev. (2023) 45:70–6. doi: 10.1016/j.braindev.2022.09.002, PMID: 36150977

[17] Okubo M Doi H Fukai R Fujita A Mitsuhashi S Hashiguchi S . GGC repeat expansion of NOTCH2NLC in adult patients with leukoencephalopathy. Ann Neurol. (2019) 86:962–8. doi: 10.1002/ana.25586, PMID: 31433517

[18] Liu YH Chou YT Chang FP Lee WJ Guo YC Chou CT . Neuronal intranuclear inclusion disease in patients with adult-onset non-vascular leukoencephalopathy. Brain: J Neurol. (2022) 145:3010–21. doi: 10.1093/brain/awac135, PMID: 35411397

[19] Chen Z Yan Yau W Jaunmuktane Z Tucci A Sivakumar P Gagliano Taliun SA . Neuronal intranuclear inclusion disease is genetically heterogeneous. Ann Clin Trans Neurol. (2020) 7:1716–25. doi: 10.1002/acn3.51151, PMID: 32777174 PMC7480908

[20] Deng J Gu M Miao Y Yao S Zhu M Fang P . Long-read sequencing identified repeat expansions in the 5’UTR of the NOTCH2NLC gene from Chinese patients with neuronal intranuclear inclusion disease. J Med Genet. (2019) 56:758–64. doi: 10.1136/jmedgenet-2019-106268, PMID: 31413119

[21] Pountney DL Raftery MJ Chegini F Blumbergs PC Gai WP . NSF, Unc-18-1, dynamin-1 and HSP90 are inclusion body components in neuronal intranuclear inclusion disease identified by anti-SUMO-1-immunocapture. Acta Neuropathol. (2008) 116:603–14. doi: 10.1007/s00401-008-0437-4, PMID: 18836734

[22] Sun QY Xu Q Tian Y Hu ZM Qin LX Yang JX . Expansion of GGC repeat in the human-specific NOTCH2NLC gene is associated with essential tremor. Brain: J Neurol. (2020) 143:222–33. doi: 10.1093/brain/awz372, PMID: 31819945

[23] Greco CM Hagerman RJ Tassone F Chudley AE Del Bigio MR Jacquemont S . Neuronal intranuclear inclusions in a new cerebellar tremor/ataxia syndrome among fragile X carriers. Brain: J Neurol. (2002) 125:1760–71. doi: 10.1093/brain/awf184, PMID: 12135967

[24] Sone J Hishikawa N Koike H Hattori N Hirayama M Nagamatsu M . Neuronal intranuclear hyaline inclusion disease showing motor-sensory and autonomic neuropathy. Neurology. (2005) 65:1538–43. doi: 10.1212/01.wnl.0000184490.22527.90, PMID: 16301479

[25] Sung JH Ramirez-Lassepas M Mastri AR Larkin SM . An unusual degenerative disorder of neurons associated with a novel intranuclear hyaline inclusion (neuronal intranuclear hyaline inclusion disease). A clinicopathological study of a case. J Neuropathol Exp Neurol. (1980) 39:107–30. doi: 10.1097/00005072-198003000-00001, PMID: 6154779

[26] Michaud J Gilbert JJ . Multiple system atrophy with neuronal intranuclear hyaline inclusions. Report of a new case with light and electron microscopic studies. Acta Neuropathol. (1981) 54:113–9. doi: 10.1007/BF00689403, PMID: 6264727

[27] Matulis SR McJunkin B Chang HH . Familial visceral neuropathy as part of a diffuse neuronal syndrome: four fatal cases in one sibship. Am J Gastroenterol. (1994) 89:792–6. 8172158

[28] Bar-Shai A Maayan C Vromen A Udassin R Nissan A Freund HR . Decreased density of ganglia and neurons in the myenteric plexus of familial dysautonomia patients. J Neurol Sci. (2004) 220:89–94. doi: 10.1016/j.jns.2004.02.017, PMID: 15140612

[29] Sone J Kitagawa N Sugawara E Iguchi M Nakamura R Koike H . Neuronal intranuclear inclusion disease cases with leukoencephalopathy diagnosed via skin biopsy. J Neurol Neurosurg Psychiatry. (2014) 85:354–6. doi: 10.1136/jnnp-2013-306084, PMID: 24039026

[30] Zhou Q Tian M Yang H Luo YB . Adult-onset neuronal intranuclear inclusion disease with mitochondrial encephalomyopathy, lactic acidosis, and stroke-like (MELAS-like) episode: A case report and review of literature. Brain Sci. (2022) 12. doi: 10.3390/brainsci12101377, PMID: 36291311 PMC9599545

[31] Yadav N Raja P Shetty SS Jitender S Prasad C Kamble NL . Neuronal intranuclear inclusion disease: A rare etiology for rapidly progressive dementia. Alzheimer Dis Associated Disord. (2019) 33:359–61. doi: 10.1097/WAD.0000000000000312, PMID: 31094708

[32] He A Wang Z Wu X Sun W Yang K Feng W . Incidence of post-stroke cognitive impairment in patients with first-ever ischemic stroke: a multicenter cross-sectional study in China. Lancet Reg Health West Pac. (2023) 33:100687. doi: 10.1016/j.lanwpc.2023.100687, PMID: 37181529 PMC10166998

[33] Liu Y Li H Liu X Wang B Yang H Wan B . Clinical and mechanism advances of neuronal intranuclear inclusion disease. Front Aging Neurosci. (2022) 14:934725. doi: 10.3389/fnagi.2022.934725, PMID: 36177481 PMC9513122

