# Supplementary material for: Neuronal intranuclear inclusion disease with initial manifestation of intractable nausea and vomiting responsive to corticosteroids: a case report
Source: Front Immunol. 2026 Mar 18;17:1782547. doi: 10.3389/fimmu.2026.1782547 (PMC13039027; doi:10.3389/fimmu.2026.1782547)
Supplement: Supplementary file 1 [file Table1.docx]

Supplementary Table 1 laboratory findings

| Laboratory test | Results | Normal references |
| --- | --- | --- |
| White Blood Cell Count (WBC) | 9.37↑ | 3.69-9.16 10^9/L |
| Neutrophil Count (NEU) | 6.25 | 2.0-7.0 10^9/L |
| Lymphocyte Count (LYM) | 2.5 | 0.8-4.0 10^9/L |
| Albumin (ALB) | 35.4 | 35.0-55.0 g/L |
| Globulin (GLB) | 20.9 | 20.0-35 g/L |
| C-reactive Protein (CRP) | 1.38 | 0.0-3.0 mg/L |
| Hemoglobin A1c (HbA1c) | 5.7 | 4.2-5.9% |
| Lactic Acid (LAC) | 2.45↑ | 0.5-2.22mmol/L |
| Homocysteine (HCY) | 9.50 | 4.0-15.4ummol/L |
| Triglycerides (TG) | 2.47 | ＜1.70 mmol/L |
| Total Cholesterol (CHOL) | 4.77 | 2.90-5.20 mmol/L |
| High-Density Lipoprotein Cholesterol (HDL) | 1.21 | ＞1.04 mmol/L |
| Low-Density Lipoprotein Cholesterol (LDL) | 2.93 | ＜3.4 mmol/L |
| Creatinine (CREA) | 59 | 53.0-115.0 umol/L |
| Urea | 4.30 | 2.90-8.20 mmol/L |
| Uric Acid (UA) | 403.0 | 89.0-430.0 umol/L |
| Glucose (GLU) | 5.83 | 3.9-6.1 mmol/L |
| Alanine Aminotransferase (ALT) | 9.3 | 0.0-40.0 IU/L |
| Aspartate Aminotransferase (AST) | 14.1 | 0.0-45.0 IU/L |
| D-dimer | 0.58 | 0.00-0.70 ug/ml |
| Free Triiodothyronine (FT3) | 3.81 | 3.10-6.80 pmol/L |
| Free Thyroxine (FT4) | 13.40 | 12.0-22.0 pmol/L |
| High-Sensitivity Thyroid-Stimulating Hormone (HTSH) | 3.11 | 0.27-4.20 uIU/ml |
| Thyroperoxidase antibody (TPOAb) | 13.7 | 0-34 IU/ml |
| Thyroglobulin antibody (TgAb) | 15.3 | 0-1155 IU/ml |
| Sodium (Na⁺) | 136.0 | 135.0-145.0 mmol/L |
| Potassium (K⁺) | 3.53 | 3.50-5.50 mmol/L |
| Chloride (Cl⁻) | 99.0 | 98.0-108.0 mmol/L |
| Calcium (Ca²⁺) | 2.25 | 2.25-2.75 mmol/L |
| Creatine kinase (CK) | 32.0 | 25.0-195.0 IU/L |
| Creatine kinase-MB (CK-MB) | 11.3 | 2.0-25.0 IU/L |
| Lactate dehydrogenase (LDH) | 167.0 | 114.0-240.0 IU/L |
| Myoglobin(MYO) | 44.10 | 0.0-70.0 ug/L |
| Vitamin B12 | 571.0 | 191-663 ng/L |
| Folate | 7.0 | 4.20-19.8 ug/L |
| Infectious disease screening | negative | negative |
| Antinuclear antibody (ANA) | negative | negative |
| Plasma catecholamines | negative | negative |
| Urinalysis | negative | negative |
| Stool routine | negative | negative |
| Coagulation profile | negative | negative |
